# Long-Term Survival and Dialysis Dependency Following Acute Kidney Injury in Intensive Care: Extended Follow-up of a Randomized Controlled Trial

**DOI:** 10.1371/journal.pmed.1001601

**Published:** 2014-02-11

**Authors:** Martin Gallagher, Alan Cass, Rinaldo Bellomo, Simon Finfer, David Gattas, Joanne Lee, Serigne Lo, Shay McGuinness, John Myburgh, Rachael Parke, Dorrilyn Rajbhandari, for the POST-RENAL Study Investigators and the ANZICS Clinical Trials Group

**Affiliations:** 1 The George Institute for Global Health, Sydney, Australia; 2 University of Sydney, Sydney, Australia; 3 Menzies School of Health Research, Darwin, Australia; 4 Austin Hospital, Heidelberg, Australia; 5 Royal Prince Alfred Hospital, Camperdown, Australia; 6 Auckland City Hospital, Auckland, New Zealand; 7 St. George Clinical School, University of New South Wales, Sydney, Australia; Mario Negri Institute for Pharmacological Research, Italy

## Abstract

**Background:**

The incidence of acute kidney injury (AKI) is increasing globally and it is much more common than end-stage kidney disease. AKI is associated with high mortality and cost of hospitalisation. Studies of treatments to reduce this high mortality have used differing renal replacement therapy (RRT) modalities and have not shown improvement in the short term. The reported long-term outcomes of AKI are variable and the effect of differing RRT modalities upon them is not clear. We used the prolonged follow-up of a large clinical trial to prospectively examine the long-term outcomes and effect of RRT dosing in patients with AKI.

**Methods and Findings:**

We extended the follow-up of participants in the Randomised Evaluation of Normal vs. Augmented Levels of RRT (RENAL) study from 90 days to 4 years after randomization. Primary and secondary outcomes were mortality and requirement for maintenance dialysis, respectively, assessed in 1,464 (97%) patients at a median of 43.9 months (interquartile range [IQR] 30.0–48.6 months) post randomization. A total of 468/743 (63%) and 444/721 (62%) patients died in the lower and higher intensity groups, respectively (risk ratio [RR] 1.04, 95% CI 0.96–1.12, *p* = 0.49). Amongst survivors to day 90, 21 of 411 (5.1%) and 23 of 399 (5.8%) in the respective groups were treated with maintenance dialysis (RR 1.12, 95% CI 0.63–2.00, *p* = 0.69). The prevalence of albuminuria among survivors was 40% and 44%, respectively (*p* = 0.48). Quality of life was not different between the two treatment groups. The generalizability of these findings to other populations with AKI requires further exploration.

**Conclusions:**

Patients with AKI requiring RRT in intensive care have high long-term mortality but few require maintenance dialysis. Long-term survivors have a heavy burden of proteinuria. Increased intensity of RRT does not reduce mortality or subsequent treatment with dialysis.

**Trial registration:**

www.ClinicalTrials.gov NCT00221013

*Please see later in the article for the Editors' Summary*

## Abstract 2

Martin Gallagher and colleagues examine the long-term outcomes of renal replacement therapy (RRT) dosing in patients with acute kidney injury randomized to normal vs. augmented RRT.

Please see later in the article for the Editors' Summary

## Editors' Summary

**Background:**

Throughout life, the kidneys perform the essential task of filtering waste products (from the normal breakdown of tissues and from food) and excess water from the blood to make urine. Chronic kidney disease (caused, for example, by diabetes) gradually destroys the kidneys' filtration units (the nephrons), eventually leading to life-threatening end-stage kidney disease. However, the kidneys can also stop working suddenly because of injury, infection, or poisoning. Acute kidney injury (AKI) is much more common than end-stage kidney disease and its incidence is increasing worldwide. In the US, for example, the number of hospitalizations that included an AKI diagnosis rose from 4,000 in 1996 to 23,000 in 2008. Moreover, nearly half of patients with AKI will die shortly after the condition develops. Symptoms of AKI include changes in urination, swollen feet and ankles, and tiredness. Treatments for AKI aim to prevent fluid and waste build up in the body and treat the underlying cause (e.g., severe infection or dehydration) while allowing the kidneys time to recover. In some patients, it is sufficient to limit the fluid intake and to reduce waste build-up by eating a diet that is low in protein, salt, and potassium. Other patients need renal replacement therapy (RRT), life-supporting treatments such as hemodialysis and hemofiltration, two processes that clean the blood by filtering it outside the body.

**Why Was This Study Done?:**

The long-term outcomes of AKI (specifically, death and chronic kidney disease) and the effects of different RRT modalities on these outcomes are unclear. A recent controlled trial that randomly assigned patients with AKI who were managed in intensive care units (ICUs) to receive two different intensities of continuous hemodiafiltration (a combination of hemodialysis and hemofiltration) found no difference in all-cause mortality (death) at 90 days. Here, the researchers extend the follow-up of this trial (the Randomized Evaluation of Normal vs. Augmented Levels of renal replacement therapy [RENAL] study) to investigate longer-term mortality, the variables that predict mortality, treatment with long-term dialysis (an indicator of chronic kidney disease), and functional outcomes in patients with AKI treated with different intensities of continuous RRT.

**What Did the Researchers Do and Find?:**

For the Prolonged Outcomes Study of RENAL (POST-RENAL), the researchers extended the follow-up of the RENAL participants up to 4 years. Over an average follow-up of 43.9 months, 63% of patients in the lower intensity treatment group died compared to 62% of patients in the higher intensity group. Overall, a third of patients who survived to 90 days died during the extended follow-up. Among the survivors to day 90, 5.1% and 5.8% of patients in the lower and higher intensity groups, respectively, were treated with maintenance dialysis during the extended follow-up. Among survivors who consented to analysis, 40% and 44% of patients in the lower and higher intensity groups, respectively, had albuminuria (protein in the urine, an indicator of kidney damage). Patients in both groups had a similar quality life (determined through telephone interviews). Finally, increasing age, APACHE III score (a scoring system that predicts the survival of patients in ICU), and serum creatinine level (an indicator of kidney function) at randomization were all predictors of long-term mortality.

**What Do These Findings Mean?:**

These findings indicate that patients with AKI in ICUs who require RRT have a high long-term mortality. They show that few survivors require maintenance dialysis for chronic kidney disease but that there is a substantial rate of albuminuria among survivors despite relative preservation of kidney function. The findings also suggest that the intensity of RRT has no significant effect on mortality or the need for dialysis. Because these findings were obtained in a randomized controlled trial, they may not be generalizable to other patient populations. Moreover, although data on mortality and maintenance dialysis were available for all the trial participants, clinical and biochemical outcomes were only available for some participants and may not be representative of all the participants. Despite these study limitations, these findings suggest that survivors of AKI may be at a high risk of death or of developing chronic kidney disease. Survivors of AKI are, therefore, at high risk of further illness and long-term albuminuria reduction strategies may offer a therapeutic intervention for this group of patients.

**Additional Information:**

Please access these websites via the online version of this summary at http://dx.doi.org/10.1371/journal.pmed.1001601.

- The US National Kidney and Urologic Diseases Information Clearinghouse provides information about the kidneys and about all aspects of kidney disease and its treatment; the US National Kidney Disease Education Program provides resources to help improve the understanding, detection, and management of kidney disease (in English and Spanish)
- The Mayo Clinic provides information for patients about acute kidney injury
- Wikipedia has a page on acute kidney injury (note that Wikipedia is a free online encyclopedia that anyone can edit; available in several languages)
- The not-for-profit UK National Kidney Federation provides support and information for patients with kidney disease and for their carers, including a link to a video about acute kidney injury
- World Kidney Day, a joint initiative between the International Society of Nephrology and the International Federation of Kidney Foundations (IFKF), aims to raise awareness about kidneys and kidney disease; its website provides information about acute kidney injury
- The MedlinePlus Encyclopedia has a pages about acute kidney failure and about renal dialysis
- The UK National Institute for Health and Care Excellence (NICE) recently published new guidelines on the treatment of acute kidney injury; a clinical practice guideline for acute kidney injury produced by KDIGO (a not-for-profit organization that aims to improve the care and outcomes of kidney disease patients worldwide through the development and implementation of global clinical practice guidelines) is available; the Acute Kidney Injury app provides a fast and simple way to explore guidelines on the diagnosis, prevention, and management of AKI

## Introduction

Acute kidney injury (AKI) is approximately ten times more common than end-stage kidney disease [1], and the incidence is increasing worldwide [2],[3]. Short-term mortality rates of patients with AKI are in excess of 40%, predominantly in those who require renal replacement therapy (RRT) [4].

The longer term outcomes of patients with AKI are less clear. Existing descriptions of these outcomes have used variable methodologies and have been obtained from retrospectively defined cohorts. Whilst some have been population-based cohorts [5],[6], many have been based upon specific disease groups [7]–[9]. In addition, the AKI cohorts are often defined using post-AKI exposure data, such as hospitalization coding [10] or based upon survival of the acute hospitalization [6]. For clinicians, when managing patients presenting with AKI, these factors limit the applicability of these studies.

Following an episode of AKI, the balance of the risks of mortality and that of subsequent chronic kidney disease (CKD) remains uncertain. A recent meta-analysis by Coca and colleagues [11] reported absolute rates of CKD following AKI approximately 50% higher than that for mortality, but was limited by a high degree of statistical heterogeneity. A large population cohort study in 2009 concluded that AKI necessitating in-hospital dialysis was associated with an increased risk of chronic dialysis but not an increase in all-cause mortality [5].

Patients with AKI managed in an intensive care unit (ICU) often require RRT and have the highest short-term mortality of any group with AKI [4]. Studies that have examined different dose intensities of RRT have not demonstrated improvements in short-term outcomes [12]. Longer term outcomes of patients treated with different intensities of RRT are unknown.

We previously conducted a randomized-controlled trial comparing higher and lower intensities of continuous RRT [13] in ICU patients with AKI and demonstrated no difference in all-cause mortality at 90 days between the two groups. The aim of this study was to extend follow-up to up to four years and report longer-term mortality (along with the variables that may predict mortality), treatment with chronic dialysis, and functional outcomes in patients treated with different intensities of continuous RRT.

## Methods

### Study Design

A description of the Randomized Evaluation of Normal vs. Augmented Levels of renal replacement therapy (RENAL) study design has been previously published [14]. In brief, it was a parallel group, open-label, randomized-controlled trial in 1,508 ICU patients with AKI requiring RRT from 35 centres in Australia and New Zealand between December 2005 and August 2008. Patients in ICU aged 18 or older, deemed by the treating clinician to require RRT and meeting at least one of the following criteria, were eligible for enrolment: oliguria (urine output < 100 ml in a 6-hour period) that was unresponsive to fluid resuscitation, serum potassium exceeding 6.5 mmol per litre, severe acidaemia (pH<7.2), a plasma urea nitrogen above 25 mmol per litre (70 mg per decilitre), a serum creatinine concentration above 300 µmol per litre (3.4 mg per decilitre), or the presence of clinically significant organ oedema (e.g., pulmonary oedema). Eligible patients were randomly assigned to receive 25 ml/kg/h (lower intensity) or 40 ml/kg/h (higher intensity) of continuous haemodiafiltration and were followed to 90 days after randomization. Study treatment was ceased when any of five pre-defined criteria were met: withdrawal of consent, death, discharge from ICU, when intermittent dialysis was considered preferable to continuous RRT for the patient, or when the treating clinicians considered that RRT was no longer required.

The Prolonged Outcomes Study of RENAL (POST-RENAL) was an investigator-initiated, prospective, extended follow-up of the RENAL study, funded by a project grant from the Australian National Health and Medical Research Council. The study was designed and managed by the study management committee and endorsed by the Australia and New Zealand Intensive Care Society Clinical Trials Group. The study protocol was approved by human research ethics committees at each of the participating centres and centrally by the Australian Institute for Health and Welfare Ethics Committee. Statistical analyses were conducted at the George Institute for Global Health.

### Study Participants

All participants in the RENAL study were included in the primary and secondary outcomes of the POST-RENAL study. Tertiary outcomes were obtained in the subset of consenting survivors. Ethics committee approval granted a waiver of the requirement for individual consent to link to state and national registries, whereupon survivors were approached for written informed consent to participate in the collection of the tertiary outcomes for the POST-RENAL study.

### Follow-up

Using the RENAL Study database, we identified all participants alive at day 90 following randomization. The initials and study numbers of these participants were provided to all the participating centres who then added patient identifiers to these data. These data were then used to link to mortality registries in all Australian states and nationally in New Zealand, along with the Australia and New Zealand Dialysis and Transplant (ANZDATA) Registry. In addition, study centres used medical records along with contact details to ascertain the survival and dialysis status of patients.

The following registries were accessed to contribute to the primary and secondary outcomes of the POST-RENAL study: Data Linkage Unit, Australian Institute of Health and Welfare National Death Index, Canberra, Australia (accessed July 2010, October 2010, January 2011, and September 2011); NZ Births, Deaths and Marriages Registry, Department of Internal Affairs, Wellington, New Zealand; Victorian Registry of Births, Deaths and Marriages, Victoria, Australia (accessed October 2010 and January 2012); Research and Statistics, Department of Justice, Queensland, Australia (accessed October 2010 and July 2011); NSW Registry Births Deaths and Marriages, New South Wales, Australia (accessed October 2010, April 2011 and October 2011); WA Registry of Births, Deaths and Marriages, Western Australia, Australia (accessed October 2010); Consumer and Business Services, Births, Deaths and Marriages Registration Office, South Australia, Australia (accessed October 2011); Births, Deaths and Marriages – ACT, Australian Capital Territory, Australia (accessed October 2011); Australia and New Zealand Dialysis and Transplant (ANZDATA) Registry, South Australia, Australia (accessed March 2011 and January 2012).

### Outcome Measures

The primary outcome measure was all-cause mortality 3.5 years after randomization. This measure was assessed by follow-up from the study centres and by linking to state and national death registries. Causes of death were independently adjudicated by two medical practitioners from death certificate data, with consensus arrived at for any discrepant classifications. The secondary outcome was treatment with maintenance dialysis (defined as entry to a chronic dialysis program and meeting criteria for inclusion in the national dialysis registry) during the 3.5 years following randomization. Information on maintenance dialysis was obtained by follow-up from the study centres and by linkage to the Australian and New Zealand Dialysis and Transplant (ANZDATA) Registry.

Tertiary outcomes were quantification of renal function in survivors determined by serum creatinine and estimated glomerular filtration rate (eGFR) using the Modification of Diet in Renal Disease Study Group (MDRD) formula [15]; prevalence of proteinuria measured by spot urinary albumin to creatinine ratio (ACR); blood pressure and receiving treatment to lower blood pressure; quality of life using the EuroQol Group 5 dimension tool (EQ-5D) [16]; and the 12 variable Short Form Health Survey (SF-12) [17]. EQ-5D derives a score after assigning the worst imaginable health state a value of 0 and the best imaginable health state a value of 1, although it does permit negative scores. SF-12 uses eight domains, with scores ranging from 0 to 100, that allow aggregation into physical health and mental health composite scores, with higher scores representing better quality of life.

Quality of life was assessed by telephone interviewers based at the George Institute for Global Health; the interviewers were blinded to original study treatment allocation.

Baseline characteristics of the study population included demographic and laboratory variables, the presence of sepsis (defined by the presence of a focus of infection and two systemic inflammatory response syndrome criteria [18] within 24 hours following randomization), the Acute Physiology and Chronic Health Evaluation (APACHE) III score (a severity of illness score ranging from 0 to 299, with higher scores indicating more severe illness [19]), and the Sequential Organ Failure Assessment (SOFA) score (ranging from 0 to 4 for each of six organ systems with higher scores indicating more severe organ dysfunction) [20].

### Statistical Analysis

The analysis followed a statistical analysis plan developed prior to any analysis of the study data (see Text S1). The dialysis-free days outcome from this plan is presented in Table S1. The data were analysed using SAS version 9.2. Where data were missing, we report the number of available observations and make no assumptions about missing values. Analyses were unadjusted, except where indicated. All tests were two-sided with a nominal value of α = 0.05. Discrete variables were summarised by frequencies and percentages; continuous variables by mean and standard deviation (SD) or median and interquartile range (IQR) where appropriate. Univariate analysis was performed using chi-square tests for binary outcomes and Student t-tests for normally distributed outcomes.

The long-term survival was analysed according to two approaches: (1) from the time of patient randomization into the RENAL study, (2) from 90 days following randomization into the RENAL study (the final follow-up point for the RENAL Study). Data were censored at the time when the patient was last known to be alive. Survival curves and estimated median survival time (if available) and 95% confidence interval were generated according to the Kaplan-Meier method. The log-rank test was used to assess the difference between the two survival curves. Mortality beyond 90 days following randomization underwent multivariate analysis using Cox modelling using backward stepwise regression for variable selection (eliminating variables with *p*-values >0.05) and forced inclusion of the RENAL study treatment variable.

As death and chronic dialysis treatment are competing risks, a competing risk sensitivity analysis was performed between the two events. This analysis was based on the cumulative incidence function (CIF) and Gray's test to test the difference between the CIFs [21].

## Results

### Study Patients

**Figure 1:**
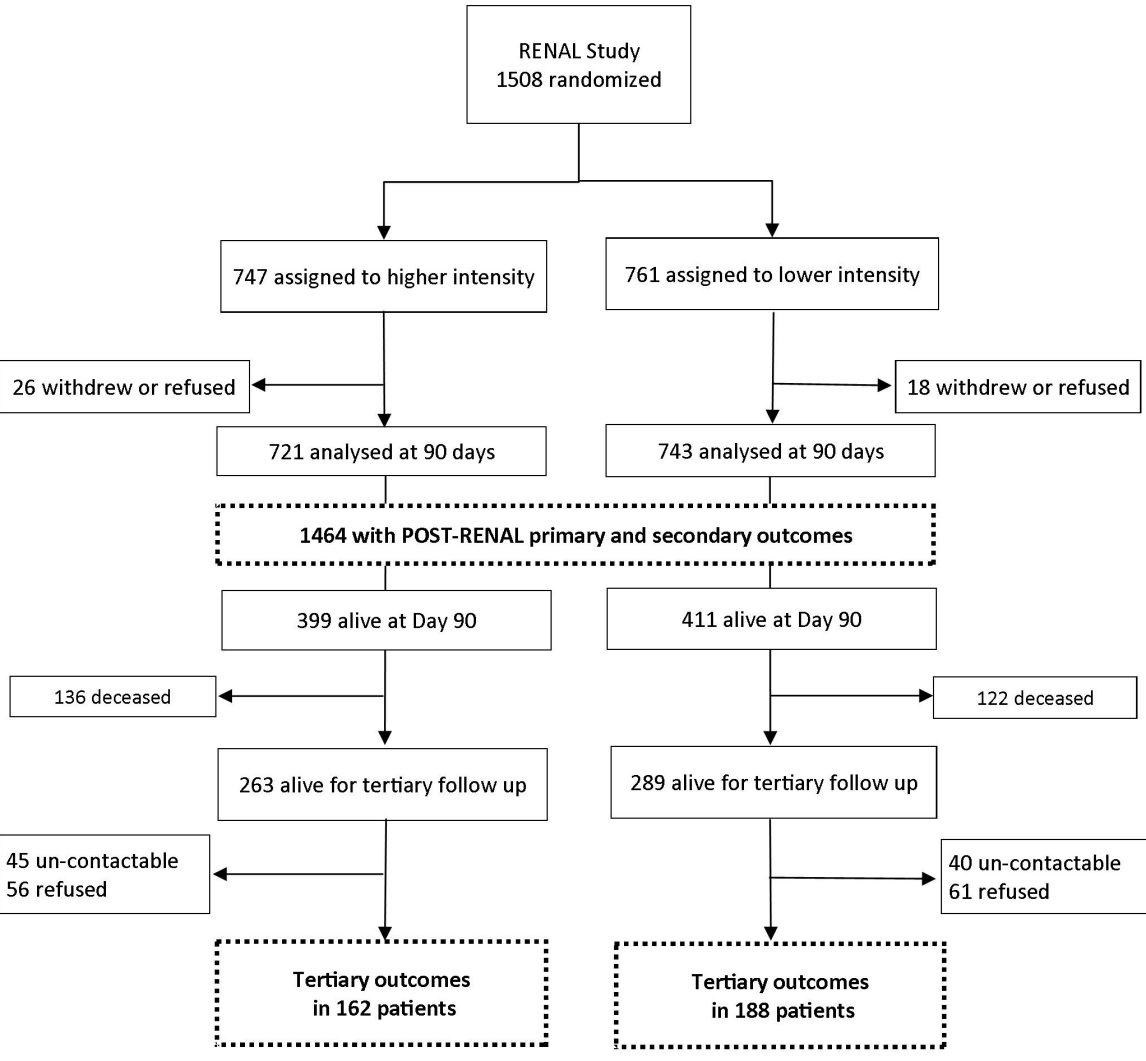
Study flow diagram. The primary outcome was mortality, and the secondary outcome was treatment with maintenance dialysis.

Of the 1,508 patients randomized, 810 patients survived to day 90 after randomization (Figure 1), with 552 of these survivors (68%) alive at the time of the POST-RENAL study. Tertiary outcomes were available on 350 (63%) of these survivors who consented to follow-up between August 2010 and January 2012.

The baseline characteristics of all RENAL Study participants have been previously reported, and those of the 810 survivors at day 90 and of the 350 consenting to clinical follow-up are presented in Table 1.

**Table 1:** Baseline characteristics by study treatment allocation of participants alive at day 90 and those consenting to clinical follow-up in the POST-RENAL study.

Characteristic	Alive at Day 90 (*n* = 810)		Consented to Clinical Follow-up (*n* = 350)	
	Lower Intensity	Higher Intensity	Lower Intensity	Higher Intensity
Number of participants	411	399	188	162
Age in years	62.5 (16)	62.9 (15)	61.3 (16)	62.2 (14)
Male sex *n* (%)	260 (63.3)	257 (64.4)	133 (70.7)	112 (69.1)
Mean preadmission eGFR^a^	58.7 (28)	52.7 (32)	59.9 (27)	56.1 (30)
Time in ICU before randomization (h, median ± IQR)	4 (18–45)	5 (18–48)	7 (24–57	6 (17–45)
Mechanical ventilation – *n* (%)	281 (68.4)	266 (66.7)	135 (71.8)	113 (69.8)
Severe sepsis – *n* (%)	177 (43.1)	191 (47.9)	81 (43.1)	84 (51.9)
APACHE III score (mean ± SD)	97.9 (24)	97 (23)	97.8 (23)	95.4 (23)
Weight – kg (mean ± SD)	81.3 (13)	81.9 (13)	81.7 (13)	83 (13)
Non-operative primary diagnosis – *n* (% of total)	279 (67.8)	294 (73.6)	123 (65.4)	115 (70.9)
Cardiovascular (*n*, % of non-op)	138 (49.4)	142 (48.3)	65 (52.8)	62 (53.9)
Genitourinary (*n*, % of non-op)	75 (26.8)	82 (27.8)	26 (21.1)	29 (25.2)
Respiratory (*n*, % of non-op)	29 (10.4)	39 (13.3)	15 (12.2)	16 (13.9)
Gastrointestinal (*n*, % of non-op)	20 (7.2)	17 (5.8)	9 (7.3)	5 (4.3)
Other (*n*, % of non-op)	17 (6)	14 (4.8)	8 (6.5)	3 (2.7)
Operative primary admission diagnoses – *n* (% of total)	132 (32.1)	105 (26.3)	65 (34.6)	47 (29)
Cardiovascular (*n*, % of operative)	87 (65.9)	70 (66.7)	43 (66.1)	30 (63.8)
Gastrointestinal (*n*, % of operative)	25 (18.9)	23 (21.9)	13 (20)	11 (23.4)
Trauma (*n*, % of operative)	9 (6.8)	5 (4.8)	4 (6.2)	3 (6.4)
Other (*n*, % of operative)	11 (8.3)	7 (6.7)	5 (7.7)	3 (6.4)
Criteria for use of RRT^b^				
Oliguria (*n*, %)	256 (62.2)	229 (57.4)	112 (59.6)	96 (59.3)
Hyperkalaemia (*n*, %)	31 (7.5)	40 (10)	11 (5.9)	15 (9.3)
Severe acidosis (*n*, %)	141 (34.3)	123 (30.8)	60 (31.9)	44 (27.2)
BUN > 25 mmol/l (*n*, %)	145 (35.3)	180 (45.1)	65 (34.3)	65 (40.1)
Creatinine > 300 µmol/l (*n*, %)	222 (54)	227 (56.9)	99 (52.7)	89 (54.9)
Severe organ oedema associated with AKI (*n*, %)	174 (42.3)	174 (43.6)	75 (39.9)	67 (41.4)
BUN (mmol/l, mean ± SD)	22.2 (12)	24.4 (13)	21.5 (11)	22.6 (13)
Creatinine before randomization (µmol/l, mean ± SD)	136 (115)	156 (117)	133 (123)	143 (88)
Bicarbonate (mmol/l, mean ± SD)	18.3 (5.9)	18.0 (5.4)	18.7 (6.3)	18.5 (5.7)

a Pre-admission renal function was only available on 433/810 (53%) of day 90 survivors of the RENAL Study.

b Percentage adds up to >100 owing to the presence of more than 1 criteria in some patients

### Outcomes

**Figure 2:**
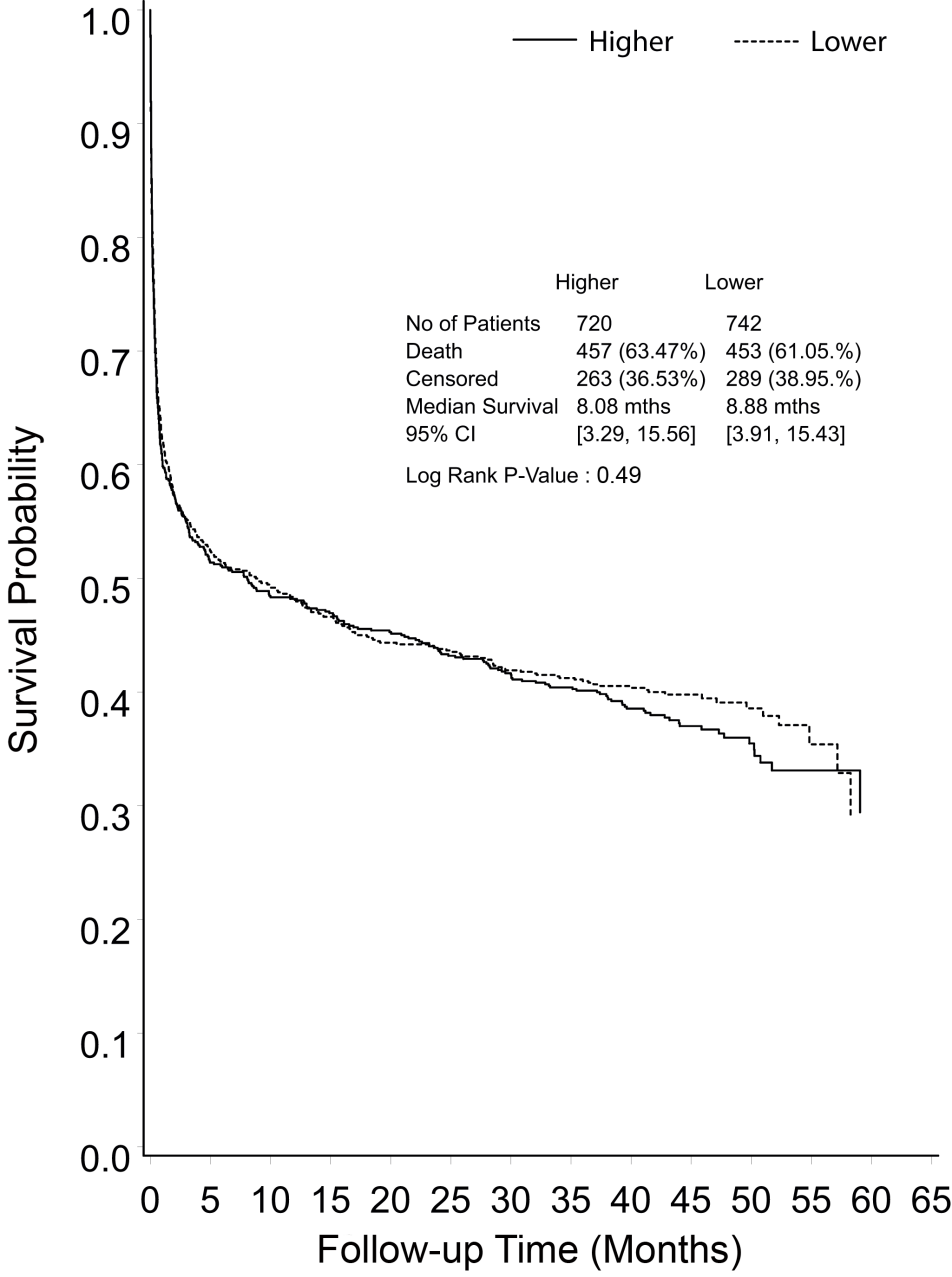
Kaplan-Meier survival curve for all study participants from randomization to end of extended follow-up, shown by treatment group.

Primary and secondary outcomes were derived at a median of 42.4 months (IQR 30.0–48.6 months) post randomization. There were a further 258 deaths during the POST-RENAL study (122 in the lower intensity group, 136 in the higher intensity group), giving an overall mortality rate of 62% in the study cohort (Figure 2).

**Figure 3:**
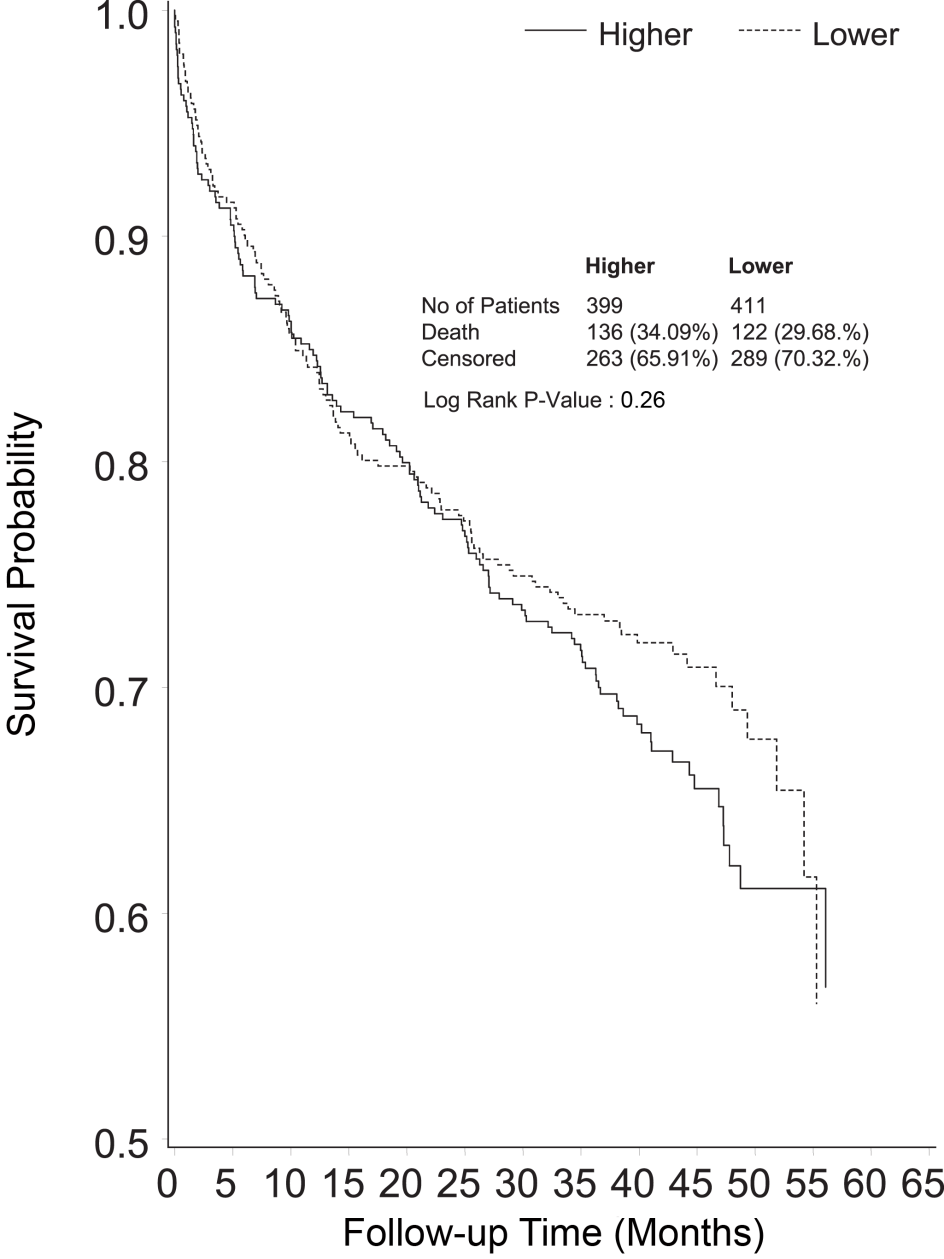
Kaplan-Meier survival curve censoring deaths before day 90 of follow-up (end point of the RENAL Study follow-up), shown by treatment group.

Median survival from randomization was 8.9 months in the lower intensity group and 8.1 months in the higher intensity group (RR 1.04, 95% CI 1.12, *p* = 0.49) (Figure 2). Excluding deaths before day 90, mortality in the lower intensity group was 122 of 411 (29.7%) and in the higher intensity group was 136 of 399 (34.1%) (RR 1.15, 95% CI 0.94–1.40, *p* = 0.26) (Figure 3). Causes of death beyond day 90 were similar in the two groups (Table 2), with more deaths in the higher intensity group ascribed to infectious causes (22 versus 35 deaths, *p* = 0.05).

**Table 2:** 

Cause of Death	Lower Intensity *N* (% of total)	Higher Intensity *N* (% of total)	*p*-Value
**Infectious causes**			0.05
Pneumonia	6 (4.9%)	19 (14%)	
Other infection/septicaemia	16 (13.1%)	16 (11.7%)	
**Cardiovascular causes**			0.47
Ischaemic heart disease	16 (13.1%)	17 (12.5%)	
Cardiac failure	12 (9.8%)	8 (5.9%)	
Other vascular disease	5 (4.1%)	10 (7.4%)	
**Malignancy**			0.12
Cancer	17 (13.9%)	29 (21.3%)	
Haematological malignancy	7 (5.7%)	3 (2.2%)	
**Metabolic**			0.24
Respiratory failure	9 (7.4%)	5 (3.7%)	
Renal failure	6 (4.9%)	6 (4.4%)	
Liver failure	7 (5.7%)	3 (2.2%)	
**Other or unknown**	21 (17.2%)	20 (14.7%)	0.58
**Total**	**122**	**136**	

*p*-Values refer to differences across the category of death by treatment allocation.

**Figure 4:**
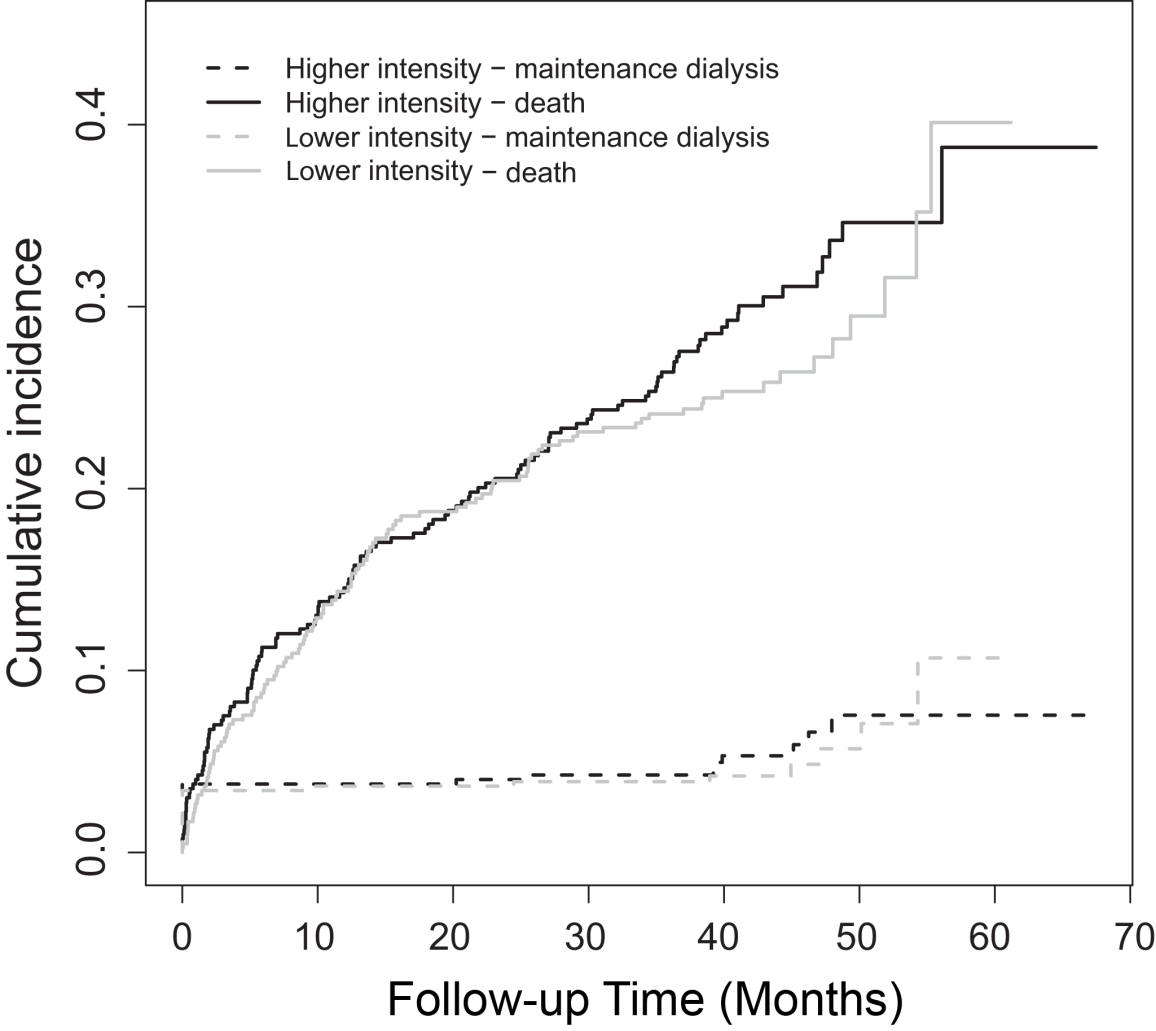
Cumulative incidence functions, comparing time to the first event of either the requirement for chronic dialysis or death beyond day 90 following randomization (each curve shown by treatment group).

Forty-four patients (5.4% of those alive at day 90) were treated with maintenance dialysis (Figure 4), 21 of 411 (5.1%) in the lower intensity group and 23 of 399 (5.8%) in the higher intensity group (RR 1.12, 95% CI 0.63–2.00, *p* = 0.69). Thirty-four of these patients (77.2%) entered the maintenance dialysis program before day 90 following randomization, and ten (22.8%) entered after day 90. The cumulative incidence of the competing outcomes of death or treatment with chronic dialysis is illustrated in Figure 4. Of the 12 patients whose death was ascribed to renal failure, two had entered a dialysis program before death.

Forty-four patients (5.4% of those alive at day 90) were treated with maintenance dialysis (Figure 4), 21 of 411 (5.1%) in the lower intensity group and 23 of 399 (5.8%) in the higher intensity group (RR 1.12, 95% CI 0.63–2.00, *p* = 0.69). Thirty-four of these patients (77.2%) entered the maintenance dialysis program before day 90 following randomization, and ten (22.8%) entered after day 90. The cumulative incidence of the competing outcomes of death or treatment with chronic dialysis is illustrated in Figure 4. Of the 12 patients whose death was ascribed to renal failure, two had entered a dialysis program before death.

The tertiary outcomes are presented in Table 3. The mean eGFR in participating survivors was 58 ml/min/1.73 m^2^ and the prevalence of micro- or macro-albuminuria was 123/292 (42.1%), the values were similar in the two study groups (*p* = 0.72, and *p* = 0.48, respectively) (Table 3). Table 4 illustrates the cross tabulation of eGFR and albuminuria based upon recent guideline recommendations [22]. The only statistically significant difference between the two study groups in any of the tertiary outcomes was a higher diastolic blood pressure in the higher intensity group. In view of the number of outcomes analysed, this finding may be due to chance alone.

**Table 3:** Clinical and biochemical outcomes in extended follow-up participants.

Outcomes	*n* Analysed	All Participants	Lower Intensity	Higher Intensity	*p*-Values
*n* blood pressure lowering medications (mean ± SD)	350	1.7 (0.9)	1.9 (1.0)	1.6 (0.8)	0.45
Systolic blood pressure (mm Hg, mean ± SD)	340	132 (18)	131 (16)	133 (20)	0.17
Diastolic blood pressure (mm Hg, mean ± SD)	339	74.8 (12)	73.4 (11)	76.3 (12)	0.02
Serum creatinine at follow-up (µmol/l, mean ± SD)	343	150 (136)	146 (120)	154 (153)	0.59
eGFR at follow-up (ml/min/1.73 m^2^, mean ± SD)	343	58 (30)	58 (29)	59 (30)	0.72
Change in creatinine from baseline to follow-up (µmol/l, mean ± SD)^a^	343	−202 (196)	−204 (207)	−200 (183)	0.87
Change in eGFR from baseline to follow-up (ml/min/1.73 m^2^, mean ± SD)^a^	343	38 (29)	38 (29)	39 (30)	0.69
Urinary ACR (mg/mmol, mean ± SD)	292	0.7 (2–7.5)	0.7 (2.4–8.7)	0.7 (1.9–6.3)	0.55
Urinary ACR ≤ 3.5 mg/mmol (*n*, %)	292	172 (58.9)	86 (56.6)	86 (61.4)	0.45
Urinary ACR > 3.5 and ≤ 35 mg/mmol (*n*, %)	292	94 (32.1)	53 (34.9)	41 (29.3)	0.28
Urinary ACR >35 (*n*, %)	292	29 (9.9)	14 (9.2)	15 (10.7)	0.68
Micro or macro-albuminuria (*n*,%)	292	123 (42.1)	67 (44.1)	56 (40)	0.48

a Difference between baseline serum creatinine and eGFR from the RENAL study (just prior to acute RRT initiation) and POST-RENAL study clinical follow-up.

**Table 4:** Prevalence of CKD by eGFR and albumin to creatinine ratio in follow-up participants.

eGFR Categories ml/min/1.73 m^2^	Urine ACR Categories (mg/mmol)		
	<3	≥3 and ≤30	>30
≥90	30	11	1
60–89	74	25	1
45–59	33	20	3
30–44	18	24	3
15–29	5	19	11
<15	2	1	12
Total	162	100	31

Two patients with an ACR performed have missing eGFR measurement.

Quality of life in participating survivors, as measured using the EQ-5D composite index, was not different between the two study groups, with mean scores of 0.8 in the lower intensity group and 0.7 in the higher intensity group (SD = 0.3, *p* = 0.70) (Table S2). The SF-12 physical composite scores (mean ± SD, lower intensity 41.1±12 versus higher intensity 49.8±11, *p* = 0.34) and the SF-12 mental composite scores (49.6±12 versus 49.4±11, *p* = 0.89) among survivors were not different between the two study treatments.

### Multivariate Predictors of Long-Term Mortality

**Figure 5:**
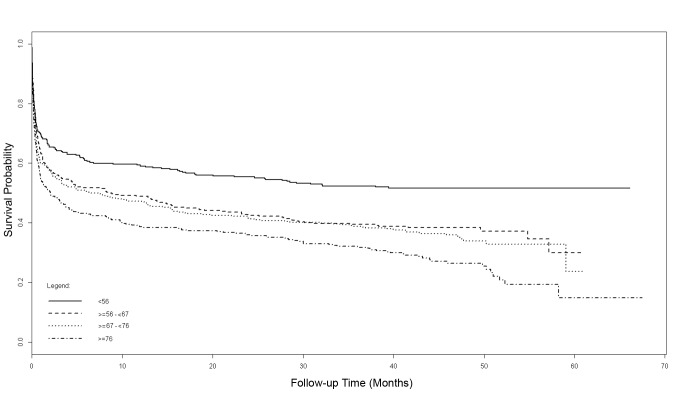
Adjusted Cox model survival curves from randomization, stratified by quartiles of age.

Results of the univariate Cox modelling of the long-term mortality from randomization are presented in Table S3. The results of the multivariate Cox models are summarized in Figure 5 and Table 5. Increasing age of participants, divided using quartiles with the lowest age group (<56 years at randomization) as the reference, revealed incremental increases in mortality with age. After adjustment for other variables in the multivariate model, patients <56 years of age at randomization had a mortality of 48% at 3.5 years, compared to 71% in those aged >76 years (Figure 5). Other baseline variables that were statistically significant predictors of mortality on multivariate modelling were APACHE III score and serum creatinine at randomization. Study treatment intensity did not influence mortality.

**Table 5:** Cox multivariate model for long-term mortality from randomization.

Variable	Comparator	Hazard Ratio (95% CI)	*p*-Value
**Age (by quartiles)**	<56 years (index)		
	56–67 years	1.39 (1.14–1.70)	0.001
	67–76 years	1.52 (1.24–1.85)	<0.001
	>76 years	1.85 (1.53–2.25)	<0.001
**Study treatment**	Higher vs lower dose	1.07 (0.94–1.22)	0.32
**APACHE III score**	10 unit increase	1.12 (1.09–1.15)	<0.001
**Randomization serum creatinine**	44 µmol/l increase	0.96 (0.94–0.97)	<0.001

## Discussion

### Statement of Key Findings

We conducted an extended follow-up of patients randomized to a large study of ICU patients requiring RRT for AKI to establish the long-term mortality rate, the need for maintenance dialysis, prevalence of renal dysfunction, and the quality of life among survivors. We found that patients with AKI treated with RRT in the ICU were at high risk of dying during the 3.5-year follow-up period; overall 31.9% of those surviving to 90 days died during the extended follow-up period. The risk of dying was much greater than the risk of entering a maintenance dialysis program, with neither outcome being influenced by the use of a higher intensity of RRT. The rate of albuminuria in survivors was substantial, despite relative preservation of renal function.

### Relationship to Existing Literature

Existing data on the long-term consequences of an episode of AKI are largely derived from administrative or disease specific cohorts [10],[23]. The absolute rates of CKD development and mortality reported from these studies have been highly variable [11], likely reflecting the different study designs and selection criteria, with pooled rates of 25.8 per 100 person years and 16.8 per 100 person years, respectively. The pooled relative risks from these studies, compared to non-AKI patients, of any CKD development following an episode of AKI was 8.8, for the development of end-stage kidney disease was 3.1, and for mortality was 2.0 [11]. The nature of these studies and their reporting has contributed to a perception that the risk of renal disease progression should be of primary concern in such patients. Our findings differ from many of these reports and these discrepancies are likely to be due to differences in the populations studied and the methodologies used. In particular, previous reports have not specifically targeted patients with AKI requiring RRT in the ICU, and such patients are at greater risk of dying compared with general hospital patients with AKI, as shown in a Swedish study of similar patients [23].

The paucity of prospective data has also limited our understanding of the natural history of AKI. Independent of eGFR, chronic proteinuria is a risk factor for death, cardiovascular disease, and the later requirement for dialysis [24],[25]. However, the prevalence of this risk factor following an episode of AKI has remained uncertain. Data from a broader population in Australia report a prevalence of proteinuria of 2.4% in the entire population that increases to 6.6% in those over 65 years of age [26]. Data from the National Health and Nutrition Examination Surveys cite a prevalence of 9.5% for albuminuria [27], and further data from a provincial laboratory registry in Canada report a prevalence of 25% using the ACR [28]. The degree of proteinuria in our study population prior to their renal injury is not known, but these other reports would suggest it is likely to be substantially less than the rate seen after the AKI in our study. Using recent classifications of CKD that include both eGFR and proteinuria to predict future risk of events [28], 13% of the survivors in our study population are in the highest risk group for death or renal functional decline.

The quality of life of survivors following treatment with RRT for AKI in the ICU has been described in a limited number of centres or shortly following the index hospitalization. Delannoy and colleagues [29] reported quality of life at six months following hospitalisation in seven centres; their results are consistent with ours, with very similar SF-12 physical and mental composite scores. When compared to a general population cohort with an eGFR < 60 ml/min/1.73 m^2^ in the Australian Diabetes, Obesity and Lifestyle Study [30], the SF-12 physical and mental component scores in our patients were similar. The EQ-5D composite scores in our severe AKI survivors most closely approximate those seen in renal transplant recipients [31].

### Implications of Study Findings

Our study highlights the increased long-term risk of death associated with AKI treated with RRT in an ICU. Only one-third of randomized patients were alive 3.5 years later, a lower survival than that seen in recognised high mortality conditions such as the acute respiratory distress syndrome [32]. Although, in our patients the risk of subsequent maintenance dialysis dependence is low, almost half have evidence of significant proteinuria, portending further risk in the years to come. These findings support the view that survivors of AKI are at increased risk and that closer surveillance may be justified. In addition, our findings suggest that chronic proteinuria reduction strategies, which have shown benefit in some patient groups with proteinuria, may warrant investigation as a therapeutic intervention.

### Study Strengths and Limitations

The strength of these findings lies in the prospective nature of the study along with the scale and completeness of long-term follow-up. The design allows for greater precision in estimates of absolute risk and enhances the clinical applicability of the findings whilst avoiding potential bias from retrospective selection of the study cohort. However, a further consequence of such a study design is that the findings from a randomized trial are not always generalizable to other patient populations, so caution should be applied in extrapolating these findings from an ICU cohort to other patients with AKI. In addition, while data linkage has allowed near complete follow-up of mortality and maintenance dialysis outcomes, the clinical and biochemical outcomes were only available for a consenting sub-group that might not be representative of the broader cohort.

### Summary

In a large cohort of patients with AKI randomized to differing doses of continuous RRT in the ICU, the increased risk of death continues well beyond hospital discharge and is not altered by increased intensity of dialysis. The proportion of patients entering a maintenance dialysis program is small but there is a high prevalence of proteinuria amongst survivors suggesting significant ongoing risk of chronic kidney disease and mortality.

## Supporting Information

Table S1 **Dialysis-free days outcome.**
(DOCX)

Table S2 **Other quality of life outcomes.**
(DOCX)

Table S3 **Univariate Cox model for mortality.**
(DOCX)

Text S1 **Statistical analysis plan.**
(DOCX)

## Abbreviations

ACR: albumin to creatinine ratio
AKI: acute kidney injury
CKD: chronic kidney disease
eGFR: estimated glomerular filtration rate
ICU: intensive care unit
IQR: interquartile range
POST-RENAL: Prolonged Outcomes Study of Randomised Evaluation of Normal vs. Augmented Levels of renal replacement therapy
RENAL: Randomised Evaluation of Normal vs. Augmented Levels of renal replacement therapy
RR: risk ratio
RRT: renal replacement therapy
SD: standard deviation
