# Supplementary material for: Long-Term Survival and Dialysis Dependency Following Acute Kidney Injury in Intensive Care: Extended Follow-up of a Randomized Controlled Trial
Source: PLoS Med. 2014 Feb 11;11(2):e1001601. doi: 10.1371/journal.pmed.1001601 (PMC3921111; doi:10.1371/journal.pmed.1001601)
Supplement: Table S1 — Dialysis-free days outcome. (DOCX) [file pmed.1001601.s001.docx]

## Dialysis-free days outcome

**Table S1**: **Mean dialysis-free days at Day 28, Day 60 and Day 90 by treatment group**

| outcomes | Lower Intensity N = 743 | Higher Intensity N = 722 | P-value* |
| --- | --- | --- | --- |
|  |  |  |  |
| Dialysis free days at Day 28 |  |  |  |
| n | 741 | 721 | 0.4038 |
| Mean, SD | 10.8 (10.1) | 10.3 (10.2) |  |
| Q1 Q2 Q3 | 0.0 11.0 21.0 | 0.0 9.0 21.0 |  |
| Missing | 2 | 1 |  |
|  |  |  |  |
| Dialysis free days at Day 60 |  |  |  |
| n | 742 | 721 | 0.2443 |
| Mean, SD | 12.0 (12.1) | 11.3 (11.9) |  |
| Q1 Q2 Q3 | 0.0 12.0 22.0 | 0.0 9.0 21.0 |  |
| Missing | 1 | 1 |  |
|  |  |  |  |
| Dialysis free days at Day 90 |  |  |  |
| n | 742 | 722 | 0.4464 |
| Mean, SD | 12.3 (13.0) | 11.7 (13.4) |  |
| Q1 Q2 Q3 | 0.0 12.0 22.0 | 0.0 9.0 21.0 |  |
| Missing | 1 | 0 |  |

T:\Statistics\Projects\Post-Renal\Outputs\Renal_dialysis_free_days.sas

Data cutoff: 08MAR2008 Last run: 01MAR2012 11:15

Assumptions in the derivation of the dialysis-free day outcomes:

1. Dialysis free days at Day 28

• If a randomized patient dies before stopping renal replacement therapy (RRT), be that the study treatment and/or dialysis = 0 dialysis free days

• If a randomized patients survives ICU, is discharged after 8 days, is off study treatment but on intermittent dialysis and dialysis continues with the last day being on day 15 after randomization = 13 dialysis free days

• If a randomized patient gets study treatment…and can stop it after 3 days..and then goes home on day 14 =25 dialysis free days

• If a patients gets RRT for 10 days..gets out of ICU without dialysis…but then on day 16 dies off dialysis = 6 dialysis free days

1. Dialysis free days at Day 60

• If patient died on Day 28 then dialysis free days at Day 28 is the same as dialysis free days at Day 60

• If patient still on dialysis at Day 28 and died after @28 in ICU then dialysis free days =0

• If patient still on dialysis at Day 28 and still on Dialysis at Day 90 then dialysis free days =0

• If patient still on dialysis at Day 28 but stopped dialysis in between but less than 32 days then Dialysis free days is the total number of days up to Day 60. For example if a patient stopped treatment at Day 40 but is on RRT for 5 extra days then the dialysis free days is at Day 60 is 60 – 45 = 15.

• If patient not on dialysis at Day 28 but had dialysis still alive then

• If Dialysis on Day 28 is not ongoing but patient is ongoing dialysis at Day 90 then dialysis free day is dialysis free days at D 60 is the same as dialysis free days at Day 28 + 29. For example if a patient is on dialysis over 15 days at day 28, i.e. dialysis free days at Day 28 is 13 days. At the end of the trial(Day 90), if the same patient is ongoing dialysis then I assume the patient was 16 days free dialysis (half of the period on Dialysis and the other half on free dialysis). Therefore the dialysis free days at day 60 for this patient is 13+16

• If patient is not ongoing dialysis at Day 28, died in between but not in ICU then dialysis free day at Day 60 is dialysis free days at Day 28 + number of days he survived between Day 28 to Day 60.

1. Dialysis free days at Day 90

• Same rules for Day 60 applied for Day 90 by using 90 days instead of 60 days
