# Supplementary material for: Long-Term Survival and Dialysis Dependency Following Acute Kidney Injury in Intensive Care: Extended Follow-up of a Randomized Controlled Trial
Source: PLoS Med. 2014 Feb 11;11(2):e1001601. doi: 10.1371/journal.pmed.1001601 (PMC3921111; doi:10.1371/journal.pmed.1001601)
Supplement: Table S2 — Other quality of life outcomes. (DOCX) [file pmed.1001601.s002.docx]

## Quality of life scores

**Table S2:** **Additional quality of life outcomes in extended follow-up participants**

| Clinical outcomes (Mean ± sd) | All N = 350 | Standard Intensity N = 188 | High  Intensity N = 162 | P-value |
| --- | --- | --- | --- | --- |
| QoL from EQ-5 questionnaire |  |  |  |  |
| EQ-5D composite score | 0.8 ( 0.3) | 0.7 ( 0.3) | 0.8 ( 0.3) | 0.7012 |
|  |  |  |  |  |
| How good is your health today (0=worst - 100=best) | 68.3 (19.7) | 68.4 (19.8) | 68.1 (19.6) | 0.9212 |

T:\Statistics\Projects\Post-Renal\Programs\a_clinical_outcomes.sas

Data cutoff: 02FEB2012 Last run: 18APR2012 16:00
