# Supplementary material for: Long-Term Survival and Dialysis Dependency Following Acute Kidney Injury in Intensive Care: Extended Follow-up of a Randomized Controlled Trial
Source: PLoS Med. 2014 Feb 11;11(2):e1001601. doi: 10.1371/journal.pmed.1001601 (PMC3921111; doi:10.1371/journal.pmed.1001601)
Supplement: Table S3 — Univariate Cox model for mortality. (DOCX) [file pmed.1001601.s003.docx]

## Univariate Cox regression of death from randomization

**Table S3: Univariate Cox regression of Death from randomization**

| Variable name | Effect (discrete variable) | Hazards ratio (95% CI) | P-values |
| --- | --- | --- | --- |
| Age by group category | [56 - 67[ vs. <56 | 1.40 (1.15 ; 1.71) | 0.0010 |
|  | [67 - 76[ vs. <56 | 1.64 (1.34 ; 2.00) | <.0001 |
|  | >=76 vs. <56 | 1.89 (1.56 ; 2.30) | <.0001 |
|  |  |  | . |
| Age |  | 1.02 (1.01 ; 1.02) | <.0001 |
|  |  |  | . |
| Gender | Male vs. Female | 0.99 (0.87 ; 1.14) | 0.9245 |
|  |  |  | . |
| Treatment | Lower vs. Higher | 0.96 (0.84 ; 1.09) | 0.4907 |
|  |  |  | . |
| Dialysis free days at Day 28 |  | 0.92 (0.91 ; 0.92) | <.0001 |
|  |  |  | . |
| Dialysis free days at Day 60 |  | 0.94 (0.93 ; 0.94) | <.0001 |
|  |  |  | . |
| Dialysis free days at Day 90 |  | 0.94 (0.94 ; 0.95) | <.0001 |
|  |  |  | . |
| Severe sepsis at baseline | Yes vs. No | 1.22 (1.07 ; 1.39) | 0.0026 |
|  |  |  | . |
| Apache III score (by 10 Unit score increase) |  | 1.13 (1.10 ; 1.16) | <.0001 |
|  |  |  | . |
| SOFA Respiration (category: Normal, Dysfunction & Failure) | Failure vs. Dysfunction | 1.38 (1.17 ; 1.64) | 0.0002 |
|  | Normal vs. Dysfunction | 0.87 (0.61 ; 1.26) | 0.4689 |
|  |  |  | . |
| SOFA Respiration (score) |  | 1.18 (1.10 ; 1.27) | <.0001 |
|  |  |  | . |
| SOFA Coagulation (category: Normal, Dysfunction & Failure) | Failure vs. Dysfunction | 1.33 (1.08 ; 1.64) | 0.0068 |
|  | Normal vs. Dysfunction | 0.88 (0.76 ; 1.01) | 0.0640 |
|  |  |  | . |
| SOFA Coagulation (score) |  | 1.14 (1.08 ; 1.20) | <.0001 |
|  |  |  | . |
| SOFA Liver (category: Normal, Dysfunction & Failure) | Failure vs. Dysfunction | 1.61 (1.29 ; 2.00) | <.0001 |
|  | Normal vs. Dysfunction | 0.87 (0.76 ; 1.01) | 0.0604 |
|  |  |  | . |
| SOFA Liver (score) |  | 1.14 (1.08 ; 1.21) | <.0001 |
|  |  |  | . |
| SOFA Cardiovascular (category: Normal, Dysfunction & Failure) | Failure vs. Dysfunction | 1.26 (1.02 ; 1.55) | 0.0289 |
|  | Normal vs. Dysfunction | 0.99 (0.76 ; 1.28) | 0.9128 |
|  |  |  | . |
| SOFA Cardiovascular (score) |  | 1.08 (1.04 ; 1.13) | 0.0003 |
|  |  |  | . |
| SOFA Renal (category: Normal, Dysfunction & Failure) | Failure vs. Dysfunction | 0.75 (0.65 ; 0.85) | <.0001 |
|  | Normal vs. Dysfunction | 0.87 (0.56 ; 1.36) | 0.5475 |
|  |  |  | . |
| SOFA Renal (score) |  | 0.88 (0.83 ; 0.94) | <.0001 |
|  |  |  | . |
| Overall SOFA score (all non-missing organ scores lumped together/5) |  | 1.42 (1.26 ; 1.61) | <.0001 |
|  |  |  | . |
|  |  | 1.37 (1.22 ; 1.53) | <.0001 |
|  |  |  | . |
| Patient had at least 1 organ failure (SOFA score 3-4) (Y/N) | Yes vs. No | 1.10 (0.72 ; 1.68) | 0.6556 |
|  |  |  | . |
| Patient had at least 1 non-renal failure (SOFA score 3-4) (Y/N | Yes vs. No | 1.47 (1.20 ; 1.81) | 0.0003 |
|  |  |  | . |
| Last Serum urea concentration |  | 1.00 (1.00 ; 1.01) | 0.1933 |
|  |  |  | . |
| Last creatinine concentration |  | 1.00 (1.00 ; 1.00) | <.0001 |
|  |  |  | . |
| INR |  | 1.11 (1.05 ; 1.16) | <.0001 |
|  |  |  | . |
| APPT |  | 1.01 (1.01 ; 1.01) | <.0001 |
|  |  |  | . |
| Haemoglobin (g/L) |  | 1.00 (0.99 ; 1.00) | 0.0222 |
|  |  |  | . |
| White cell count (x10^9^/L) |  | 1.00 (1.00 ; 1.01) | 0.6689 |
|  |  |  | . |
| Platelet count(x10^9^/L) |  | 1.00 (1.00 ; 1.00) | 0.0003 |
|  |  |  | . |
| Sodium (mmol/L) |  | 1.01 (1.00 ; 1.02) | 0.1072 |
|  |  |  | . |
| POTASSIUM (mmol/L) |  | 0.99 (0.93 ; 1.06) | 0.8465 |
|  |  |  | . |
| Chloride (mmol/L) |  | 1.00 (0.99 ; 1.00) | 0.2915 |
|  |  |  | . |
| Bicarbonate (mmol/L) |  | 1.00 (0.99 ; 1.01) | 0.7428 |
|  |  |  | . |
| Urea (mmol/L) |  | 1.00 (1.00 ; 1.01) | 0.1419 |
|  |  |  | . |
| Creatinine (44µmol/L, equivalent to 0.5mg/dl) |  | 0.96 (0.94 ; 0.97) | <.0001 |
|  |  |  | . |
| Phosphate (mmol/L) |  | 1.07 (0.99 ; 1.15) | 0.0869 |
|  |  |  | . |
| Albumin (g/L) |  | 0.99 (0.98 ; 0.99) | 0.0017 |
|  |  |  | . |
| Magnesium (mmol/L) |  | 1.19 (1.00 ; 1.41) | 0.0547 |
|  |  |  | . |
| pH |  | 0.43 (0.26 ; 0.71) | 0.0011 |
|  |  |  | . |
| PaCP2 (mm/Hg) |  | 1.01 (1.00 ; 1.01) | 0.0115 |
|  |  |  | . |
| Base excess (mmol/L) |  | 1.00 (0.99 ; 1.01) | 0.3338 |
|  |  |  | . |
| iCa++ (mmol/L) |  | 1.16 (0.88 ; 1.52) | 0.2839 |
|  |  |  | . |
| Glucose (mmol/L) |  | 0.99 (0.97 ; 1.01) | 0.3539 |
|  |  |  | . |
| Mechanical ventilation | Yes vs. No | 1.36 (1.17 ; 1.59) | <.0001 |
|  |  |  | . |
| Glomerular filtration rate (eGFR) |  | 1.00 (1.00 ; 1.00) | 0.5663 |
|  |  |  | . |
| Glomerular filtration rate (eGFR) < 45ml/min | Yes vs. No | 1.08 (0.91 ; 1.28) | 0.4019 |
|  |  |  | . |
| Glomerular filtration rate 45 < (eGFR) < 60ml/min | Yes vs. No | 0.91 (0.73 ; 1.13) | 0.3992 |
|  |  |  | . |
| Glomerular filtration rate (eGFR) > 60 ml/min | Yes vs. No | 1.01 (0.85 ; 1.20) | 0.8740 |
|  |  |  | . |

T:\Statistics\Projects\Post-Renal\Programs\a_Cox_FU_Death.sas

Data cutoff: 02FEB2012 Last run: 23NOV2012 10:10
