# Supplementary material for: Long-Term Survival and Dialysis Dependency Following Acute Kidney Injury in Intensive Care: Extended Follow-up of a Randomized Controlled Trial
Source: PLoS Med. 2014 Feb 11;11(2):e1001601. doi: 10.1371/journal.pmed.1001601 (PMC3921111; doi:10.1371/journal.pmed.1001601)
Supplement: Text S1 — Statistical analysis plan. (DOCX) [file pmed.1001601.s004.docx]

## Statistical Analysis Plan for the POST-Renal Study

This statistical analysis plan was finalized and agreed upon by the POST-RENAL Study Investigators on 7^th^ February 2012, with all statistical analysis of the locked study dataset occurring after this date.

### Study synopsis

| Study Title | Prolonged Outcomes Study of the RENAL Study (POST-RENAL) Study |
| --- | --- |
| Study Rationale | Acute Kidney Injury (AKI) is an increasingly common condition which is associated with high mortality. In survivors the outcomes are poorly understood with some registry studies suggesting that rates of subsequent kidney failure are high. There are no large scale prolonged follow up studies to define the longer term consequences of AKI. POST-RENAL will extend the follow up of the largest randomized trial in the field to describe these outcomes. |
| Study Design | Cohort study |
| Subject Participation | Single visit followed by single telephone survey |
| Rationale for Number of  Subjects | Survivors of randomized trial at approximately 3.5 years following randomization |
| Approximate Duration of Study | 18 months |
| Study Outcomes | Primary: Mortality  Secondary: Requirement for renal replacement therapy, Presence of markers of kidney disease and dysfunction, Quality of life. |
| Criteria for Inclusion | Survivors at 90 days following randomization in the RENAL Study |
| Criteria for Exclusion | Died within 90 days following randomization in the RENAL Study |
| Number of Subjects | 810 survivors at 90 days |
| Number of Study Centres | 35 Intensive Care Units that participated in the RENAL Study |
| Treatment Administration | Nil treatment administered |
| Safety Evaluation | As a cohort study with no interventions few safety issues are envisaged. Adverse events will be reported and monitored by the study Steering Committee. |
| Statistical Analysis, interim and final | There will be no interim analysis. The final analysis will compare the primary and secondary outcomes using log rank test, Students t test, Chi squared and the Mann-Whitney U test as appropriate. Odds ratios will be estimated using multivariate logistic regression analysis. |

### Background and rationale

The incidence of Acute Kidney Injury (AKI) is increasing with overall rates exceeding that of end-stage kidney disease by a factor of 15-fold[^1-3^](#_ENREF_1). In its most severe form, where patients require dialysis in an ICU, the mortality is over 50%[^3^](#_ENREF_3)^,^[^4^](#_ENREF_4). There are no treatments proven to reduce this mortality and there is little long term follow up data from large studies of survivors from an episode of severe AKI.

Beyond the mortality of AKI, the long term renal outcomes of survivors of AKI remain unclear. One small case series[^5^](#_ENREF_5) suggested relative preservation of renal function in survivors of AKI but there is very little data beyond hospital discharge. The VA/NIH trial reported very high rates of renal failure and dialysis dependence in the surviving patients, with 75% of survivors showing no recovery of renal function by day 28[^6^](#_ENREF_6). In addition a recent publication linking hospital admissions data with the US Renal Data System Registry [^7^](#_ENREF_7) suggests that patients with AKI had a 13 fold increase in the risk of developing end stage kidney disease over the following 2 years. This risk rose to 42 fold if the patient had chronic kidney disease in addition to an episode of AKI. These recent studies of large numbers of patients raise very serious concerns about the long term renal outcomes of AKI. However, the evidence is limited by the small numbers, short follow up and reliance upon admission coding data. There is no prolonged follow up assessment of a large cohort of patients with AKI from a randomized controlled multi-centre study.

The RENAL Study, an NHMRC funded multi-centre RCT, is the largest study of AKI treatment and randomized 1508 patients to two different dialysis dose intensities in 35 centres across Australia and New Zealand. It completed follow up on 97.1% of randomized patients out to 90 days[^8^](#_ENREF_8). There were no differences in patient mortality between the normal and augmented intensity dialysis groups and the mortality in both groups was 45%. The large numbers of patients enrolled in the RENAL Study, the demonstrated completeness of data and the absence of such follow up from the literature represent an unprecedented opportunity to define the long term outcomes of AKI in a unique manner.

This study will extend the follow up of survivors from the RENAL Study to a median of 3.5 years to provide high quality evidence regarding the long term consequences of an episode of severe AKI which represents a serious gap in our knowledge. The follow up will focus upon the mortality rates along with the frequency of markers for future renal and cardiovascular disease such as blood pressure, renal function, urinary abnormalities and quality of life.

### Study objectives

Primary Objective

The primary objective of the POST-RENAL Study is to compare long-term overall survival of adult patients with acute renal failure according to treatment allocation in the RENAL study.

Secondary Objectives

The secondary outcomes will include:

1. Dialysis free days (for all patients at the start of RENAL study, for patients alive at Day 28, for patients alive at Day 60 and patients alive at Day 90)
2. Time to first return to dialysis beyond Day 90 (death censored)
3. Causes of death, competing risk of dialysis/Death
4. Blood pressure and requirement for treatment for high blood pressure
5. Renal function: as measured by serum creatinine and eGFR
6. Proteinuria, as measured by urine albumin:creatinine ratio
7. Quality of life: as measured by two measures (EQ-5D and SF-12)

In this study, adverse events are likely to be extremely rare due to lack of a study intervention beyond data collection. However it is possible for adverse events to arise from the interactions required for participation in the study (eg. resulting from procedure of blood collection) and these will be reportable to the coordinating centre and local ethics committee where applicable.

1. Number of events and number and proportion of patients experienced serious adverse events

### Study design

This study is a cohort study, deriving extended follow up of all survivors of an earlier randomized controlled trial (the RENAL Study). The outcomes of the RENAL Study at 90 days post randomization has been ascertained on 97.1% of participants with 55% of these patients surviving.

### Study population

The study population is that of all patients alive at 90 days following randomization in the RENAL study (the primary outcome of that study). Accordingly, the POST-RENAL study will seek to operate in the same centres. The RENAL Study randomized 1508 patients in 35 centres in Australia and New Zealand.

The outcomes of the RENAL Study at 90 days post randomization has been ascertained on 97.1% (n=1464) of participants with 55% (n = 810) of these patients surviving. The first patient was enrolled in the study in December 2005 and the last in August 2008. We will follow up all patients alive at 90 days following randomization at median 3.5 years (+/- 6 months) post randomization.

Follow up will be sought on all survivors from July 2010. Where the patient has died beyond the 90 day follow up of the study, limited data on important events (requirement for dialysis, date of death, cause of death) will be sought. Those who are still alive at the time of extended follow up will be asked to consent to the full data collection.

### Statistical analysis

General principles

1. There are no treatments associated with POST-RENAL, as it constitutes an extended follow up of survivors from a previous randomized trial. However all primary and secondary outcomes will be analysed as according to original randomization in the RENAL Study as well as a cohort.
2. All tests are two-sided and the nominal level of alpha will be 5%.
3. All statistical analyses will be unadjusted except where indicated.
4. Subgroup analyses will be carried out irrespective of whether there is a significant effect of treatment on the primary outcome.
5. We will not impute missing values unless specified otherwise. We will report the number of observations used in the analysis.
6. P-values will not be adjusted for multiplicity. However the outcomes are clearly categorized by degree of importance (primary, secondary and tertiary) and a limited number of subgroup analyses are pre-specified.

Baseline characteristics of patients

Description of the following baseline characteristics will be presented by treatment group. Data for Day 90 survivors will be extracted from RENAL study database. Discrete variables will be summarized by frequencies and percentages. Percentages will be calculated according to the number of patients for whom data are available. Where values are missing, the denominator (which will be less than the number of patients assigned to the treatment group) will be stated in either the body or a footnote in the corresponding summary table. In some instances, additional frequencies and percentages of patients in each category will be reported as indicated in the list below. Continuous variables will be summarized by use of standard measures of central tendency and dispersion using mean and standard deviation as well as quantile points at 0.25, 0.5 and 0.75 where appropriate.

1. Sex
2. Age
3. Weight
4. Percentage of patients with measured weight, percentage of patients with estimated weight and method of weight estimation.
5. Source of admission to ICU (emergency department, hospital floor, another ICU, another hospital, operating room (OR) following emergency surgery, OR following elective surgery, readmission to the same ICU during same hospitalization)
6. Operative or non-operative admission
7. Operative admission diagnosis (number and % in categories a-k)
   1. Cardiovascular
   2. Respiratory
   3. GIT
   4. Neurological
   5. Trauma without traumatic brain injury
   6. Traumatic brain injury +/- multiple trauma
   7. Burns
   8. Renal
   9. Gynecology
   10. Other orthopedic
   11. Other surgical
8. Non-operative admission diagnosis (number and % in categories a-l)
   1. Cardiovascular
   2. Respiratory
   3. GIT
   4. Neurological
   5. Sepsis
   6. Trauma without traumatic brain injury
   7. Traumatic brain injury +/- multiple trauma
   8. Metabolic
   9. Haematological
   10. Burns
   11. Renal
   12. Other medical
9. Severe sepsis at baseline
10. APACHE III score
11. SOFA score
    1. Cardiovascular domain
    2. Respiratory domain
    3. Hepatic domain
    4. Haematologic domain

(SOFA score domains will be analyzed as continuous variables and as categorical variables divided into normal function (SOFA score 0) dysfunction (SOFA score 1-2) and failure: SOFA score 3-4).

1. Last serum urea concentration before randomization
2. Last serum creatinine concentration before randomization
3. Haematological variables
4. Biochemical variables
5. Treatment with mechanical ventilation
6. Estimated glomerular filtration rate (eGFR)
7. Presence of an estimated glomerular filtration rate (eGFR) <60 ml/min

The eGFR will be calculated using the revised modification of diet in renal disease (MDRD) equation[^9^](#_ENREF_9):

eGFR = 175 × (SCr x 0.0113)^-1.154^ × (age)^-0.203^ ×((0.742 [if female]))

SCr is serum creatinine level in micromol/L

Primary analysis

The primary outcome of mortality will be reported as a survival analysis from the time patients are randomized into the RENAL study. Survival curves and estimated median survival time and its 95% confidence interval will be generated according to the Kaplan-Meier method. Data will be censored at the time when the patient was last known to be alive or to be discharged from hospital. Log-rank test will be used to assess the difference between the two survival curves.

Multivariate Cox model will be performed to provide an adjusted long term survival curve of survivors of acute kidney injury. To determine which variables to include in the multivariate Cox model, we will run first a univariate Cox regression on each baseline variable and select variables with a p-value <= 0.01.

The number of events and the median survival (if available including 95% CI) will also be reported.

Secondary analysis

Time-to-event (Time to first return to dialysis beyond Day 90, Time-to-specific cause of death) will be analysed using the same principles as in the primary outcome. Because the two events Death and First return to dialysis are competing events, data will be censored at the time of competing event if it occurs first. Log-rank test will be used to assess the difference between the two survival curves.

As this cause-specific analysis can potentially be biased due to the presence of different cause of death, a competing risk analysis will also be undertaken if the number of competing events (Death and return to dialysis) occurring is moderate to large (e.g. > 10%). This could typically be based on the CIF and the Gray’s test.

For binary secondary outcomes (i.e. Requirement for treatment for high blood pressure), a standard Chi-square test will be used to assess the effect of treatment. Frequencies and percentages per arm, possibly a relative risk (or odds-ratio) measuring the treatment effect and its 95% CI will also be reported along with the p-value of the chi-squared test.

Continuous secondary outcomes (i.e. time to free requirement for dialysis, Blood pressure, Renal function: as measured by serum creatinine and eGFR and Proteinuria, as measured by urine albumin:creatinine ratio) will be summarized by use of mean and standard deviation as well as quantile points at 0.25, 0.5 and 0.75 reported. Comparison in differences in mean between the two groups will be carried out using univariate/multivariate linear regression.

Quality of life: as measured by two measures (EQ-5D and SF-12)

Descriptive statistics of individual responses to the 5 EQ-5D questions and to the 12 SF-12 questions by treatment allocation will be provided. Statistics will include the difference in mean score between the two group.

### Tables and Figures

Table 1 : Baseline characteristic (to RENAL study patients to POST-RENAL study patients)

Table 2: Time-to-event outcomes

Table 3: Binary and continuous outcomes

Figure 1 : Kaplan Meier graph of survival time from original randomization in the RENAL

Figure 2 : Kaplan Meier graph of Time to first return to dialysis beyond Day 90 (stratified by the original randomization)

Figure 3 : Kaplan Meier graph of Time to Death for Day 90 survivors

Figure 4 : Estimates of probabilities of Death and first return-to-Dialysis based on cumulative incidence functions

### References:

**1.** Ali T, Khan I, Simpson W, et al. Incidence and outcomes in acute kidney injury: a comprehensive population-based study. *J Am Soc Nephrol.* Apr 2007;18(4):1292-1298.

**2.** Waikar SS, Curhan GC, Wald R, McCarthy EP, Chertow GM. Declining mortality in patients with acute renal failure, 1988 to 2002. *J Am Soc Nephrol.* Apr 2006;17(4):1143-1150.

**3.** Bagshaw SM, George C, Bellomo R. Changes in the incidence and outcome for early acute kidney injury in a cohort of Australian intensive care units. *Crit Care.* 2007;11(3):R68.

**4.** Ympa YP, Sakr Y, Reinhart K, Vincent JL. Has mortality from acute renal failure decreased? A systematic review of the literature. *Am J Med.* Aug 2005;118(8):827-832.

**5.** Liano F, Felipe C, Tenorio MT, et al. Long-term outcome of acute tubular necrosis: a contribution to its natural history. *Kidney Int.* Apr 2007;71(7):679-686.

**6.** Palevsky PM, Zhang JH, O'Connor TZ, et al. Intensity of renal support in critically ill patients with acute kidney injury. *N Engl J Med.* Jul 3 2008;359(1):7-20.

**7.** Ishani A, Xue JL, Himmelfarb J, et al. Acute Kidney Injury Increases Risk of ESRD among Elderly. *J Am Soc Nephrol.* January 1, 2009 2009;20(1):223-228.

**8.** Bellomo R, Cass A, Cole L, et al. Intensity of continuous renal-replacement therapy in critically ill patients. *N Engl J Med.* Oct 22 2009;361(17):1627-1638.

**9.** Levey AS GT, Kusek J, et al. Simplified equation to predict glomerular filtration rate from serum creatinine. *J Am Soc Nephrol.* 2000;11:A828.
